# Improving Chemical Autoencoder Latent Space and Molecular *De Novo* Generation Diversity with Heteroencoders

**DOI:** 10.3390/biom8040131

**Published:** 2018-10-30

**Authors:** Esben Jannik Bjerrum, Boris Sattarov

**Affiliations:** 1 Wildcard Pharmaceutical Consulting, Zeaborg Science Center, Frødings Allé 41, 2860 Søborg, Denmark; 2 Science Data Software LLC, 14914 Bradwill Court, Rockville, MD 20850, USA; brois475@gmail.com

**Keywords:** deep learning, RNN, LSTM, de novo molecule design, molecular autoencoders, molecular heteroencoders, molecular data augmentation

## Abstract

Chemical autoencoders are attractive models as they combine chemical space navigation with possibilities for de novo molecule generation in areas of interest. This enables them to produce focused chemical libraries around a single lead compound for employment early in a drug discovery project. Here, it is shown that the choice of chemical representation, such as strings from the simplified molecular-input line-entry system (SMILES), has a large influence on the properties of the latent space. It is further explored to what extent translating between different chemical representations influences the latent space similarity to the SMILES strings or circular fingerprints. By employing SMILES enumeration for either the encoder or decoder, it is found that the decoder has the largest influence on the properties of the latent space. Training a sequence to sequence heteroencoder based on recurrent neural networks (RNNs) with long short-term memory cells (LSTM) to predict different enumerated SMILES strings from the same canonical SMILES string gives the largest similarity between latent space distance and molecular similarity measured as circular fingerprints similarity. Using the output from the code layer in quantitative structure activity relationship (QSAR) of five molecular datasets shows that heteroencoder derived vectors markedly outperforms autoencoder derived vectors as well as models built using ECFP4 fingerprints, underlining the increased chemical relevance of the latent space. However, the use of enumeration during training of the decoder leads to a marked increase in the rate of decoding to different molecules than encoded, a tendency that can be counteracted with more complex network architectures.

## 1. Introduction

Autoencoders have emerged as deep learning solutions to turn molecules into latent vector representations as well as decode and sample areas of the latent vector space [1,2,3]. An autoencoder consists of an encoder which compresses and changes the input information into a code layer and a decoder part which recreates the original input from the compressed vector representation (the latent space vector). After training, the encoder can be extracted from the autoencoder and used to calculate vector representations of the molecules. These can be used as a sort of molecular fingerprints or GPS for the chemical space of the molecules. The decoder can be used to translate back from the latent representation to the molecular representation used during training, such as simplified molecular-input line-entry system (SMILES). This makes it possible to use the decoder as a steered solution for molecular de novo generation, as the probability outputs of the decoder can be sampled, creating molecules which are novel but close to the point in latent space. Alternatively, the molecules of the nearby latent space can be explored by adding a suitable amount of random noise to the vector.

Various encoder–decoder architectures have been proposed as well as the encoder output functions have been regularized or manipulated using variational autoencoders [1] and adversarial autoencoders [2]. Both convolutional neural networks (CNNs), as well as recurrent neural networks (RNNs) have been used for the encoder part [1,2,3], whereas the decoder part has mostly been based on RNNs with either gated recurrent units (GRU) [4] or long short-term memory cells (LSTM) [5] to enable longer range sequence memory. The various approaches and their use in both de novo and quantitative structure activity relationship (QSAR) applications in drug discovery are part of a recent mini-review [6].

A famous painting of René Magritte, “The Treachery of Images”, shows a pipe, and also has the text “Ceci n’est pas une pipe”: This is not a pipe. The sentence is true as it is a painting of pipe, not the pipe itself, kindly reminding us that representation is not reality. Autoencoders based on SMILES strings [7] face the same fundamental issue. Is the latent space a representation of the molecules or is it a condensed representation of the SMILES strings representing the molecules?

**Figure 1:**
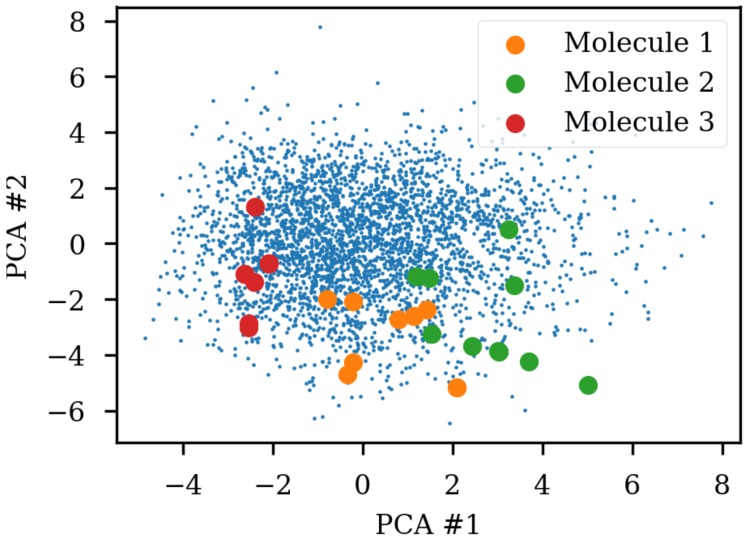
Enumeration challenge of a sequence to sequence model trained on canonical SMILES (simplified molecular-input line-entry system). The non-canonical SMILES of the same molecule is projected to different parts of the latent space reduced to two dimensions with principal components analysis (PCA). The small blue dots are the test set used for fitting the PCA. Some clustering of the enumerated SMILES can be observed.

Due to the SMILES language rules, multiple different SMILES can represent the same molecule. This has been exploited as data augmentation with the SMILES enumeration technique [8]. A simple challenge with different SMILES representations of the same molecule shows that the same molecule end up in different parts of the latent space due to the specific SMILES form used. Figure 1 shows the same three molecules after projecting into the latent space of an RNN to RNN autoencoder. The different SMILES representation of the molecules end up being projected to very different areas of the latent space, although some clustering can be observed. The latent space thus also contains information about the specific SMILES string and not only the molecule it represents which has also previously been noted [2,6]. One way of solving this challenge could be to use special engineered networks and graph based approaches [9] for molecular generation.

**Figure 2:**
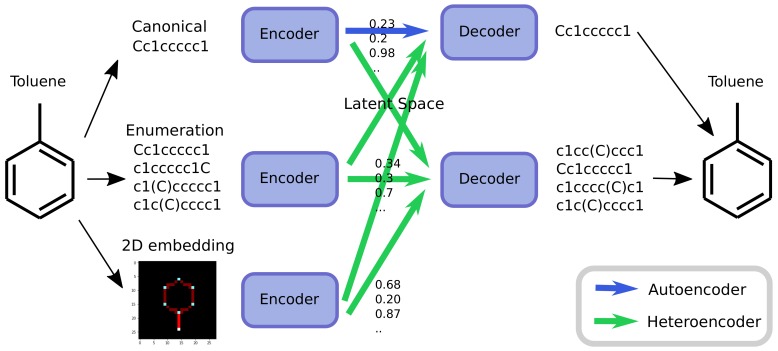
Chemical heteroencoders are similar to autoencoders but translates from one representation of the molecule to the other. The molecule toluene can be represented as a canonical SMILES strings, in different enumerated SMILES or via a 2D embedding. The autoencoder converts the canonical SMILES string to the latent space and back again (blue arrow), whereas many more possibilities exists for heteroencoders (green arrows).

As an alternative to engineering the outcome, it is here suggested that it is possible to use SMILES enumeration or chemception image embedding [10] to create chemical heteroencoders. The concept is illustrated in Figure 2. By translating from one format or representation of the molecule to the other, the encoder–decoder network is forced to identify the latent information behind both representations. This should in principle lead to a more chemically relevant latent space, independent of the representations or canonicalization used.

Here, the choice of representation and enumeration is explored for both training the encoder or decoder and the latent space similarity to SMILES and scaffold based metrics calculated. Moreover, it is tested if these changes influence the properties of the decoder when used for de novo design of molecules. Furthermore, an optimized and expanded heteroencoder architectures trained on ChEMBL23 datasets are used to encode latent vectors for subsequent use as input to QSAR models of five different molecular datasets.

## 2. Results

### 2.1. GDB-8 Dataset Based Models

All models were trained to full convergence and obtained similar train and test loss function values, the latter listed in Table 1. The models trained on enumerated SMILES output have a markedly larger final loss, but all models show a low degree of malformed SMILES when sampling the latent space vectors calculated from the test set.

#### 2.1.1. Molecular and Sequence Similarity

**Figure 3:**
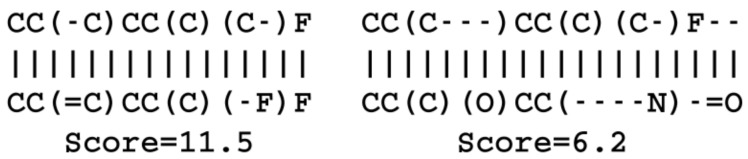
Examples of optimal SMILES alignments of a molecule with two other molecules. The score is +1 for character match, −1 for mismatch, gap openings −0.5 and gap extension −0.05. Gaps are show with dashes, “-”, and are not SMILES single bonds.

**Figure 4:**
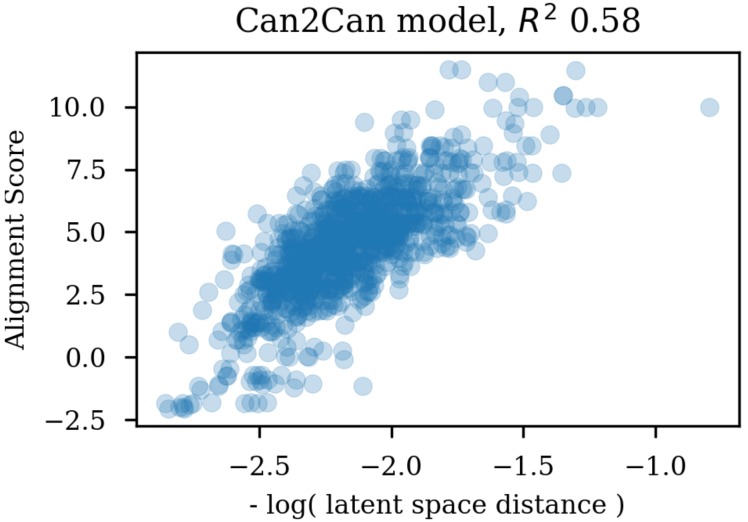
Scatter plot of the latent space similarities and the alignment scores of the SMILES strings.

**Figure 5:**
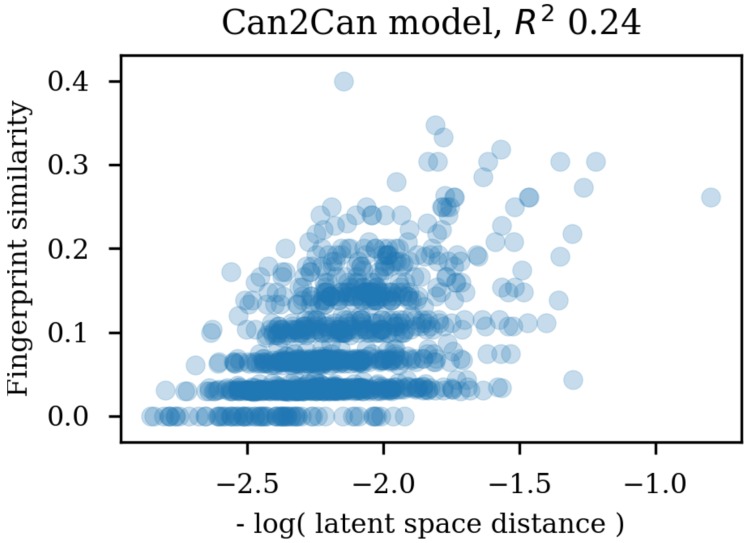
Scatter plot of the latent space similarities and the circular fingerprint similarities.

Using the same reference molecule, similarity metrics were calculated based on the latent space vectors of the test set, Morgan fingerprints and sequence alignment scores, followed by calculation of the correlation coefficients (R${}^{2}$). Examples of SMILES alignments are shown in Figure 3 for two different alignments. Figure 4 shows an example scatter plot of the alignment scores and latent space similarity for the first molecule and the rest of the molecules in the test set. The correlation between the same latent space similarity measurement and the Morgan fingerprint similarity was intended as a metric of the scaffold similarity independent of the SMILES strings, and an example scatter plot is shown in Figure 5. Both the sequence alignment score and the fingerprint based similarity have a correlation with the latent space similarity, which shows that the latent space is at least somehow related to our traditional understanding of similarities between molecules. The properties and the correlations of all the models trained on the GDB-8 dataset are listed in Table 1. The models with a decoder trained on canonical SMILES show a markedly larger correlation between the latent space and the SMILES sequence similarity metric than between the fingerprint based similarity and the latent space. In contrast, the fingerprint and sequence similarities correlations to the latent space similarity are more on the same level when the decoder is trained using enumerated SMILES. The heteroencoder based on the image embedding of the molecule has the lowest correlations, indicating a markedly different or noisy latent space.

**Figure 6:**
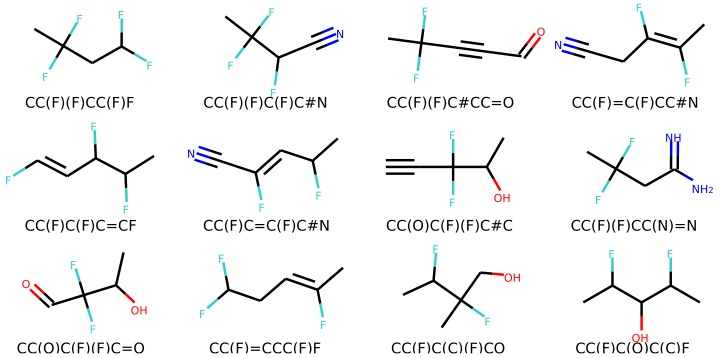
Molecules similar in latent space using the can2can model. The reference molecule is in the upper left corner and similarity drops row-wise in normal reading direction.

**Figure 7:**
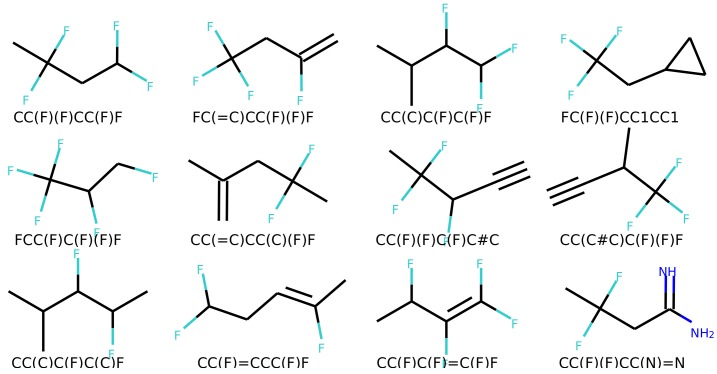
Molecules similar in latent space using the can2enum model. The reference molecule is in the upper left corner and similarity drops row-wise in normal reading direction.

Figure 6 and Figure 7 show a result of similarity searching in the latent space of the test set using a query molecule. The molecules from the can2enum model seems qualitatively more similar than the ones that are most similar in the latent space produced by the can2can model. There is overlap between the two sets, so in some respects the two latent spaces are related.

#### 2.1.2. Error Analysis

**Figure 8:**
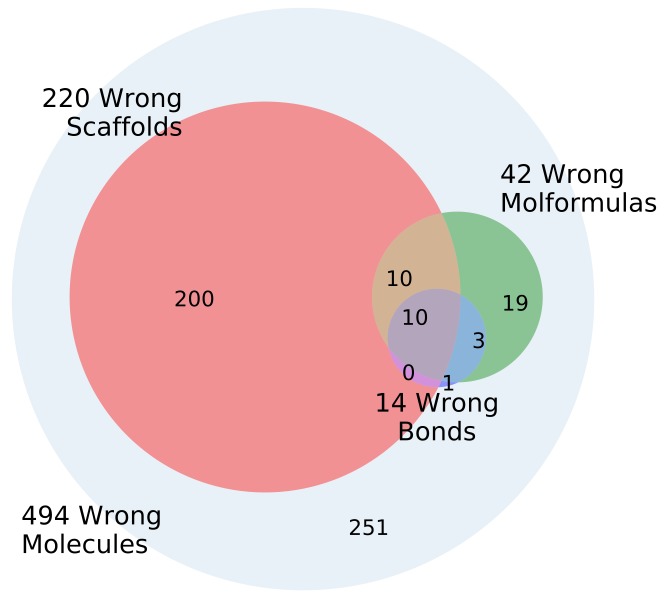
Venn diagram of the errors encounted during molecule reconstruction of 1000 molecules for the GDB-8 can2enum model.

The models in general produce large percentages of valid SMILES (Table 1). However, using enumeration in the input and output significantly increases the percentage of the outputs where the decoded molecule is not the same as the encoded molecule. The input and output molecules were further compared with regard to scaffold, molecular sum formula and equality of bonds. Figure 8 shows the error types and overlap for the can2enum model. In addition, 494 molecules out of 1000 tested were valid SMILES but not the same as the input molecule. An additional 220 had the wrong scaffold, 42 the wrong sum formula and 14 the wrong bondtypes or number of bonds. The majority (251) had the right scaffold, the right atoms and the correct bonds, but had seemingly assembled the molecule in a wrong order. The bond types and atoms are in principle simple accounting operations independant of the SMILES enumeration, whereas the models struggle more with the scaffold reconstruction and the atom order, which are influenced by the SMILES enumeration. The results for the other heteroencoder models are qualitatively similar (not shown).

#### 2.1.3. Enumeration Challenge

**Figure 9:**
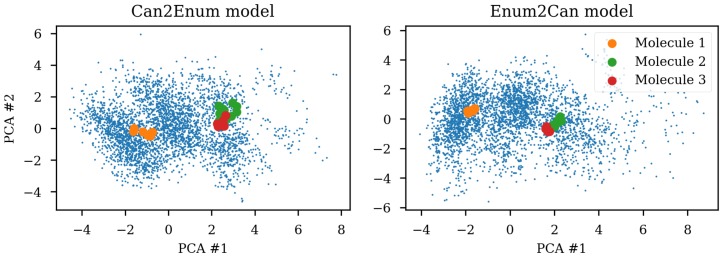
SMILES enumeration challenge of the GDB-8 dataset based Enum2can and Can2enum encoders. The same three molecules were encoded from 10 enumerated SMILES and projected to the latent space reduced to two dimensions with principal components analysis (PCA). Using enumerated SMILES for training of the encoder leads to the tightest clustering, but also training with the enumerated SMILES in the decoder improves the clustering (c.f. Figure 1). Small blue dots are the test set used for the PCA reduction.

The encoders capabilities to handle different SMILES from the same molecule were tested by projection to a PCA reduction of the latent space (c.f. Figure 1). Figure 9 shows the improvement that can be obtained by training the heteroencoders with enumerated SMILES for either the encoder and decoder. Training the encoder with enumerated SMILES strings gives the tightest clustering (enum2can), showing that the encoder has learned to recognize the same molecule independent on actual serialization of the SMILES string. By showing multiple different SMILES strings to the encoder during training, the encoder can produce the latent space coordinate most suitable for recreating the SMILES form of the decoder, irrespective of the SMILES form shown to the encoder. The enum2enum model has a similar tight clustering as the enum2can model (not shown). The can2enum model also shows more tight clustering than the can2can model from Figure 1, indicating that the heteroencoding itself changes the latent space although the encoder itself was not trained on different SMILES forms. Alternatively, the model is doing a more complicated task which could work as regularization leading to better generalization.

#### 2.1.4. Sampling Using Probability Distribution

**Figure 10:**
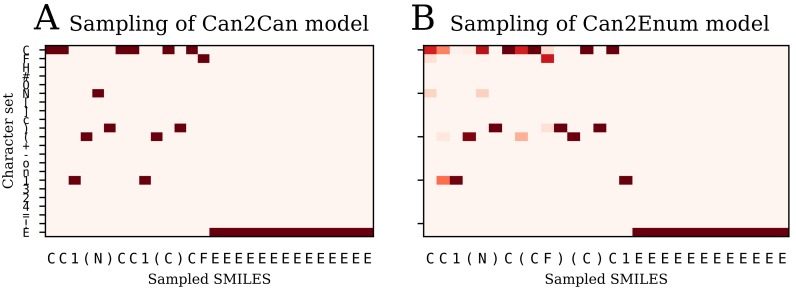
Multinomial sampling of the decoder for two different models illustrated with heat maps of the character probability for each step during decoding of the latent space. (**A**) the can2can model is very certain at each step and samples the same canonical SMILES each time; (**B**) the can2enum model has more possibilities at each step in the beginning. The probability heatmap and sampled SMILES will be different for each sampling run, depending on which character is chosen from the probability distribution at each step.

**Figure 11:**
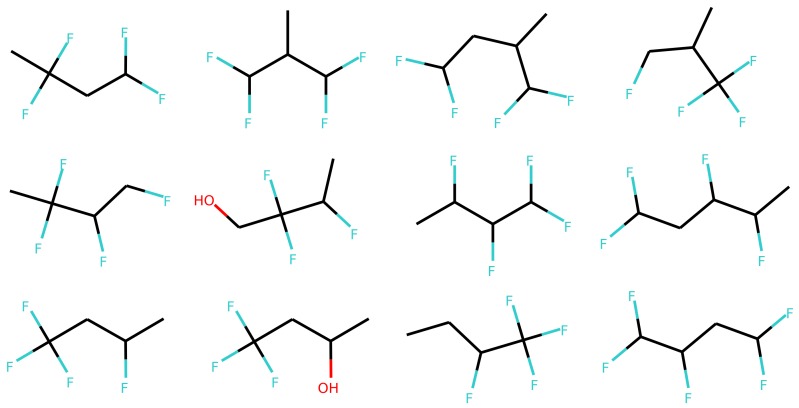
Examples of different sampled molecules using multinomial sampling with the decoder from the two layer LSTM model (enum2enum 2-layer). The one in the upper-left corner is the reference molecule used to encode the latent space coordinates.

Figure 10 illustrates the difference between probability sampling of the can2can and can2enum model. The decoder outputs a probability distribution at each step, which can be sampled randomly according to the probabilities (Multinomial sampling). For the can2can model, there is little difference between this sampling strategy and the simple selection of the most probable next character. The can2enum model instead show a lot more uncertainty in the next characters in the beginning of the sampling. The first character is most likely “C”, but also “N” and “F” are possibilities. As the model samples “C”, the next character is either a “C”, a branching “(“, or start of a ring “1”. Because it then samples “C”, it has to choose a ring start next. Towards the end of sampling, the decoder gets completely certain with the last six characters, probably because there is only one way to finish the molecule with the already sampled characters. Table 2 shows some statistics on the sampled molecules using the latent coordinates from a single molecule. The model trained on canonical SMILES in both encoder and decoder are very sure about the SMILES it want to recreate, as only one SMILES form and one molecule is sampled. In contrast, the decoders trained with the enumerated SMILES create different SMILES forms of the correct molecule, but also creates other molecules as well. The more complex model with two LSTM layers handles the task a bit better than the single layer version, which only produce the molecule presented for the encoder 20% of the times. Examples of the sampled molecules from the two layer model are shown in Figure 11.

### 2.2. QSAR Modelling Using ChEMBL Trained Heteroencoders

**Figure 12:**
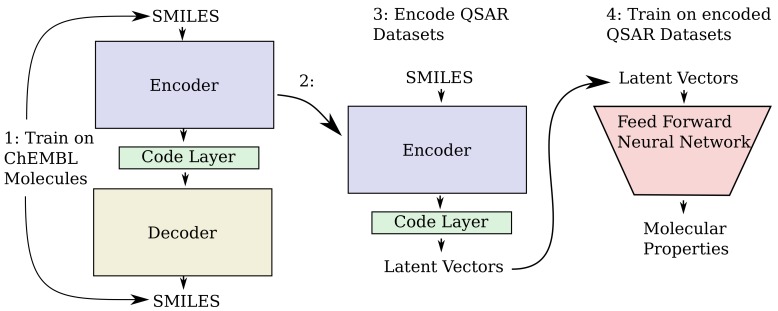
The steps used in the modelling of the QSAR dataset. In step 1, the auto-/heteroencoder is trained on a large unlabelled dataset of molecules from ChEMBL. After training, the encoder part is extracted in step 2 and used to encode the molecules from the QSAR datasets into their latent vectors in step 3. In step 4, a separate standard feed forward neural network is used to build QSAR models from training sets which are subsequently tested with the held-out test set.

The overall steps for producing the QSAR models is illustrated in Figure 12. Training of the encoder–decoder models used on the ChEMBL datasets, resulted in final losses of approximately 0.001, 0.01, 0.10, 0.11 for the can2can, enum2can, can2enum and enum2enum configurations of the training sets, respectively. Reconstruction performance of the different encoder/decoder configurations is presented in the Table 3. The performance of the QSAR modelling done based on ECFP4 fingerprints and the different vectors obtained from the code layers of the three different neural network models are shown in Table 4. There is some improvement from the ECFP based baseline models to the can2can vector based models, with further improvement for the models based on the latent vectors encoded with the heteroencoders. The three different heteroencoders seem to produce latent vectors which perform very similar to each other in the QSAR modelling, with a tendency for the average performance to rise from enum2can to enum2enum over can2enum.

An interesting observation was that approximately 40% of the neurons of the code layer for each configuration are never activated. This is likely related to the use of the ReLU activation function and diminishing or enlarging the code layer resulted in almost the same percentage of inactive neurons (results not shown). The latent vector is thus even denser than the chosen number of neurons.

## 3. Discussion

Changing the representations used for training autoencoders (here called heteroencoders) have a marked influence on the properties and organization of the latent space. Although a perfect correlation to the standard fingerprint similarity is not wanted or expected, it is more reassuring that the dependence to the SMILES sequences is at a similar level to the fingerprint based similarity, than the situation where the correlation to the SMILES sequence is much larger than the correlation to the fingerprint metric. The greater balance between the two correlations strongly indicates that the latent space is just as relevant for the molecular scaffold as it is to the SMILES sequence in itself.

The dataset used in the first part of this study was of a limited size and molecular complexity (only eight atoms). Additionally, as the dataset is fully enumerated, the same graph structures are very likely present in both training and test set, which could be the basis of the excellent reconstructions of the test sets. The model could in principle memorize all graph structures instead of learning the rules behind the graph scaffolds, and then simply assign a specific sequence of atoms to the memorized graph. Even though the dataset was somewhat simple, the models trained on enumerated data may have struggled because of a low neural network fitting capacity. This indeed seems to be the case as the 2-layer enum2enum model has much lower final loss (Table 1) and also much better reconstruction statistics when reconstructing (Table 1) and sampling the molecules (Table 2) than the single layer enum2enum model. There is a rough correlation between the SMILES validity rate and the molecule reconstruction error rat. However, with heteroencoders, the molecule reconstruction error rate becomes a more relevant term to measure than the SMILES validity rate, as the former can become high, while the SMILES validity error rate is still low.

Models employed in other studies are more complex with larger and multiple layers [1,2,3,9,10,12]. The heteroencoder concept was thus further expanded to also handle ChEMBL datasets. The expansion of the networks to two layers in both the encoder and decoder, use of bidirectional layers in the encoder and a larger number of LSTM cells allowed to fit the larger molecules, although the uncertainty in the reconstruction of the molecule is still present (c.f. Table 3). It is likely that even more complex architectures with three LSTM layers or a further enlargement of the number of LSTM cells would be needed to lower the molecule reconstruction error further.

The image to sequence model seems to be an outlier in comparison with the SMILES based models, in the respect that the latent space don’t show much correlation with neither the SMILES sequence to be decoded or the molecular graph. However, the model produces a very low percentage of invalid SMILES and also has a low error rate with respect to molecule reconstruction, but is also decoding to canonical SMILES which is an easier task than decoding to enumerated SMILES. The various other tests showed no big difference or benefit when compared to the much simpler use of different SMILES representations. However, the success of the image to SMILES model to transcode between the image and the SMILES representation illustrates that the concept is not limited to SMILES based models. Other types of presenting molecules to neural networks, such as graph convolutional approaches for molecules [13,14] would also be worth exploring for the encoder network. The heteroencoder architecture may be useful for architectural experiments with large unlabeled datasets to find better architectures and suitable deep learning feature extractions for training on molecules. The identified architectures and trained weights may be useful for transfer learning in for example QSAR modeling.

The failure of the image to SMILES heteroencoder to produce significantly better latent representation fits with the observation that the latent space is mostly influenced by the decoding procedure, not the encoding procedure. The various encoders, whether based on images, canonical SMILES or trained on enumerated SMILES, seem to learn to recognize the molecules anyway and create a latent space that is best suitable for recreation of the decoders form. It thus seems that using enumeration techniques or other molecular representation for the decoder will influence the latent space the most.

Training autoencoders on enumerated or different data further seems to improve the latent space with respect to its relevance for QSAR modelling. This is encouraging as it suggests that the encoded vectors are not only relevant for reconstruction of the molecular scaffold in itself, but additionally capture the variations underlying biological as well as physico-chemical properties of the molecules. It seem that already the encoder independence of the SMILES form for the enum2can leads to a more smooth latent space (c.f. Figure 9 panel A), which increases the relevance for QSAR modelling. This is in contrast to the results in Table 1, where a less skewed correlation to the decoded SMILES serialization in the encoder part is forced by training on enumerated data in the output, which however only leads to marginal gains in QSAR model performance.

The improvement seems quite marked and larger than what other studies have found. Winter et al. also used the heteroencoder approach in parallel to our work and found improvements for QSAR modelling [15]. However, the improvements over baseline models were not as marked as in our results. The differences in network architectures (our use of bidirectional layers, LSTM vs. GRUs and batch normalization as example) and maybe also the choice of training data (Drug like molecules of ChEMBL) could be possible explanations. Future benchmarking on common datasets will likely show the way to the best network architecture and what unlabelled datasets to use for specific tasks.

On the other hand, the solubility dataset we used have previously been carefully modelled with chosen features and topological descriptors, resulting in a R^2^ of 0.92 and a standard deviation of prediction of the test set of 0.6 [16]. Likewise, a carefully crafted QSAR model of BCF obtained a R^2^ of 0.73 and an RMSE of 0.69 [17], which is on par with our model using the can2enum derived latent vectors. However, a later benchmark showed better performance for the CORAL software for prediction of BCF (R^2^: 0.76, RMSE: 0.64) [18], suggesting that further improvements are possible.

Thus, the QSAR models based on heteroencoder derived latent vectors seem to almost match the performance of highly optimized QSAR models from selected features (c.f. Table 4), and it may rather be the ECFP4 and can2can model derived latent vectors that are mediocre for the tested type of QSAR tasks. Furthermore, the ECFP fingerprints and auto-/heteroencoder derived latent vectors are of different dimension and nature. The fingerprints are 1024-dimensional, but binary, where the latent vectors are 256-dimensional and real valued. To make sure that the improvements were not due to different optima of the model hyper parameters for the different data, the neural network architectures for the QSAR models were optimized based on the ECFP4 fingerprint input. Some improvement of the fingerprint based models were observed, but reusing the ECFP4 hyper parameters for the latent vector based modelling still resulted in a large improvement in model performance for these input types. Further tuning of the hyper parameters of the models based on the latent vectors could likely further increase the performance to some degree (not tested). On the other hand, the denser dimensionality (256 < 1024) could help protect against over fitting and make the choice of hyper parameters less critical for these models. Either way, the use of heteroencoder derived latent vectors seem to be the better choice.

Feature generation for a dataset of chemical structures using the ChEMBL trained auto-/ heteroencoders described in the publication is publicly available and hosted on the open sciende data repository (OSDR) platform [19], where it is possible to encode molecular datasets into the latent vector space for subsequent uses, such as in QSAR modelling.

The increased relevance of the latent space with respect to bioactivity and physico-chemical properties are likely to increase the relevance and quality of the de novo generated libraries where the neighborhood of as example lead compounds are sampled on purpose. However, the use of enumeration for training the decoder comes at the cost of greater uncertainty in the decoding, at a marginal improvement to the relevance of the latent space for QSAR modelling when compared to the enum2can model. On the other hand, the greater uncertainty and “creativity” in decoding could be beneficial and further help in creating more diversity in the generated libraries, but if this is the case has yet to be investigated. The choice of enumeration for decoder and/or encoder will thus likely depend on the intended use-cases.

## 4. Materials and Methods

### 4.1. Datasets

#### 4.1.1. GDB-8

The GDB-8 dataset [20,21] was downloaded and split randomly into a train and test set using a 0.9 to 0.1 ratio.

#### 4.1.2. ChEMBL23

Structures were extracted from the ChEMBL23 database [22] and validated using in-house rules at Science Data Software LLC (Rockville, MD, USA) (salts were stripped, solvents removed, charges neutralized and stereo information removed). The maximum available length of the canonical SMILES string allowed for a molecule was 100 characters. In addition, 10,000 molecules were selected randomly for the held out test set. From the remainder of the 1.2 million molecules, a training set of 400,000 molecules and a validation set of 300,000 molecules was randomly selected for use during training procedures.

#### 4.1.3. QSAR Datasets

Five experimental datasets were used, spanning physico-chemical properties as well as bioactivity. Four datasets (IGC50, BCF, MP, LD50) for QSAR modeling were downloaded from the EPA Toxicity Estimation Software Tool [23] webpage [24] and used as is without any additional standardization. The solubility was obtained from the supplementary information of [16]. The parsed dataset is availble for download [25]. Information of the datasets are shown in Table 5.

Datasets from EPA’s TEST suite were already split into train/test sets in a 75/25% ratio and were used accordingly. Molecules for the solubility dataset were obtained by resolving CAS numbers from the supporting info [16] and the dataset was randomly split using the same ratio as the other QSAR datasets.

### 4.2. 1D and 2D Vectorization

SMILES were enumerated and vectorized with one-hot encoding as previously described [8]. In addition, 2D vectorization was done similar to the vectorization used in Chemception networks [10] with the following modifications: a PCA with three principal components was calculated on atomic properties from the mendeleev python package [29] (dipole_polarizability, electron_affinity, electronegativity, vdw_radius, atomic_volume, softness and hardness). The PCA scores were normalized with min-max scaling to be between zero and one to create the atom type encoding. PCA and scaling were performed with the Scikit-Learn python package [30]. RDKit [11] was used to compute 2D coordinates and extract information about atom type and bond order. The normalized PCA scores of the atom types were used to encode the first three layers and the bond order was used to encode the forth layer. A fifth layer was used to encode the RDKit aromaticity perception. The 2D coordinates of the RDKit molecule were rotated randomly up to +/− 180° around the center of coordinates before discretization into numpy [31] floating point arrays.

### 4.3. Neural Network Modeling for GDB-8 Dataset

Sequence to Sequence RNN models were constructed using Keras v. 2.1.1 [32] and Tensorflow v. 1.4 [33]. The overall architecture follows the encoder -> code layer -> decoder scheme shown in Figure 2 with a detailed scheme in the Supplementary Information Figure S1.

The first layer consisted of 64 LSTM cells [5] used in batch mode. The final internal memory (C) and hidden (H) states were concatenated and used as input to a dense layer (the code layer) of 64 neurons with the rectified linear unit activation function (ReLU) [34]. Two separate dense layers with ReLU activation functions were used to decode the code layer outputs into the initial C and H states for the RNN based decoder. The decoder consisted of a single layer of 64 LSTM cells trained with teacher forcing [35] in batch mode. The output from the LSTM cells was connected to a Dense layer with a softmax activation function matching the dimensions of the character set. A two-layer model was also constructed by increasing the number of LSTM cells to 128 and the number of LSTM layers to two in both the encoder and decoder. Accordingly, four separate dense networks were used to decode the code layer into the initial C and H states for the two LSTM layers in the decoder.

The networks were trained with mini-batches of 256 sequences for 300 epochs using the categorical cross entropy loss function and the Adam optimizer [36] with an initial learning rate of 0.05. The two layer model was trained with an initial learning rate of 0.01. The loss was followed on the test set and the learning rate lowered by a factor of two when no improvement in the test set loss had been observed for 5 epochs.

After training in batch mode, three models were created from the parts of the full model. A decoder model from the initial input to the output of the cdoe layer. A model to calculate the initial states of the LSTM cells in the decoder, given the output of the code layer. Lastly, a stateful decoder model was constructed by creating a model with the exact same architecture as the decoder in the full model, except the LSTM cells were used in stateful mode and the input vector reduced to a size of one in the sequence dimension. After creation of the stateful model, the weights for the networks were copied from corresponding parts of the trained full model.

The image to sequence model CNN encoder was built from three different Inception-like modules [37] similar to the Chemception modelsChemception networks [10]. The architecture is shown schematically in the Supplementary Information Figure S2. The first module consisted of a tower with a 1×1 2D convolutional layer (Conv2D) followed by a 3×3 Conv2D, a tower with a 1×1 Conv2D layer followed by a 5×5 Conv2D layer and a tower with just a single 1×1 Conv2D layer. The outputs from the towers were concatenated and sent to the next module.

The standard inception module was constructed with a tower of 1×1 Conv2D layer followed by a 3×3 Conv2D layer, a tower with a 1×1 Conv2D layer followed by a 5×5 Conv2D layer, an extra tower of a 1×1 Conv2D layer followed by a 7×7 Conv2D but with only half the number of kernels and a tower with a 3×3 Maxpooling layer followed by a 1×1 Conv2D layer. All strides were 1×1. The outputs from the four towers were concatenated and sent to the next module.

The inception reduction modules were similar to the standard module, except they had no 7×7 tower and used a stride of 2×2.

A standard inception module was stacked with a reduction inception module three times, giving 7 inception modules in total including the initial one. The number of kernels was set to 32.

The outputs from the last inception module were flattened and followed by a dropout layer with a dropout rate of 0.2. Lastly the output was connected to the code layer consisting of a dense layer with the ReLU activation function. The decoder part was constructed similar to the sequence to sequence models described above with one layer LSTM cells. The image to sequence model was trained similar to the sequence to sequence models for 200 epochs.

The models are named after the training data in a encoder2decoder naming scheme. “Can” is training data with canonical SMILES, where “Enum” designates that the input or output was enumerated during the training. “Img” shows that the data was the 2D image embedding.

### 4.4. Similarity Metrics

SMILES strings sequence similarities were calculated as the alignment score reported by the pairwise global alignment algorithm of the Biopython package [38]. The match score was set to 1, the mismatch to −1, the gap opening to −0.5 and the gap extension to −0.05. The fingerprint similarity metric was calculated on basis of circular Morgan fingerprints with a radius of 2 as implemented in the RDKit library [11]. The fingerprints were hashed to 2048 bits and the similarity calculated with the RDKit packages FingerprintSimilarity function. The latent space similarity between two molecules was calculated as the negative logarithm to the Euclidean distance of the vector coordinates.

### 4.5. Enumeration Challenge

The encoder was used to calculate the latent space of the test set, followed by a dimensionality reduction with standard principal components analysis (PCA) as implemented in the Scikit-Learn package [30]. Three molecules were converted to different SMILES strings with SMILES enumeration [8]. The latent space coordinates of the non-canonical SMILES were calculated with the encoder and transformed and projected onto the visualization of the principal components from the PCA analysis.

### 4.6. Error Analysis of Output

The percentage of invalid SMILES was quantified as the number of produced SMILES which could not be validated as molecules by RDKit. Subsequently the equality of the input and output RDKit molecules was checked. The similarity of the scaffold was checked by comparing the generalized murcko scaffolds [39] including side-chains. The atom equivalence was checked by comparing the molecular sum formulas. The number and nature of bonds was compared via a “bond sum formula” by counting the number of single, double, triple and aromatic bonds.

### 4.7. Multinomial Sampling of Decoder

Multinomial sampling was implemented as previously described [12]. The sampling temperature was kept at 1.0.

### 4.8. Neural Network Modelling for the ChEMBL Dataset

The overall steps for producing the QSAR models is illustrated in Figure 12. The sequence to sequence autoencoder used for encoding the ChEMBL data and encoding of vectors for QSAR modelling was programmed in Python 3.6.1 [40] using Keras version 2.1.5 [32] with the tensorflow backend [33]. A detailed scheme of the network is available in the Supplementary Information Figure S3. The encoder consisted of two bidirectional layers of 128 CuDNNLSTM cells in each one-way layer. The final C and H states were concatenated and passed as input to a dense layer with 256 neurons using the ReLU [34] activation function (the code layer). The output from the dense layer were decoded by four parallel dense layers with the ReLU activation function, whose outputs were used to set the initial C and H states of the decoder LSTM layers. The decoder itself consisted of two unidirectional layers of 256 CuDNNLSTM cells each. The decoder was trained under teacher forcing as described for the simpler networks above. Every non-linear activation was followed by Batch Normalization. No additional regularization was used. Furthermore, 400,000 random structures from the CheMBL23 training set were pre-enumerated 50-times for each SMILES string. The new 20 million pairs were shuffled and used in both a canonical to enumerated and an enumerated to canonical setting and trained until model convergence. The same 400,000 canonical SMILES were also used to train an auto encoder from canonical to canonical SMILES. For the enumerated to enumerated training setting 50 pairs (when possible) of different SMILES strings were created for each molecule of the training set. The network was trained using mini-batches of 256 one-hot encoded SMILES strings, using the Adam optimizer with an initial learning rate of 0.005. The training was monitored and controlled by three callbacks. One callback monitored the loss of the validation set and lowered the learning rate by a factor two when no improvement had been observed for two epochs (ReduceLROnPlateu). Another Callback stopped training when no improvement in the validation set loss had been observed for five epochs (EarlyStopping), and the last callback saved the model if the validation loss has improved (CheckPoint). Models typically converged after approximately 40 epochs, which usually took about six hours on a NVIDIA GTX 1080 Ti equipped server.

### 4.9. QSAR Modelling

Subsequent QSAR modelling was performed using the machine learning capabilities of the Open Science Data Repository [41]. An initial search for hyper parameters was performed after converting the molecules into ECPF4 fingerprints (radius 2, 1024 bits). The hyper parameter search for a neural network was performed using Tree of Parzen Estimators (TPE) algorithm [42] as implemented in Hyperopt [43] with the search space bounds listed in Table 6. The performance on each dataset was optimized using 3-fold cross validation on the training set. The performance of the model with the final hyperparameters were subsequently tested on the held-out test set using an ensemble of 10 models build during 10-fold cross validation during training. The auto-/heteroencoders trained on the ChEMBL23 molecules were subsequently used to encode the QSAR datasets into vectors using the output from the code layer. The vectors were used as input to the QSAR models. The same hyper parameters were used as identified for the ECFP4 based models, with no further attempt to optimize the hyper parameters of the feed forward neural networks using the auto-/heteroencoder encoded molecules.

## 5. Conclusions

The pilot study using a fully enumerated train and test set with eight atoms showed that the latent space representation is sensitive to the chosen representations of the input and output in the training. Using canonical SMILES for the decoder gives a latent space representation which seems closer correlated to the SMILES strings than to the molecular graphs. In contrast, training the encoders on input and output from different representations in chemical heteroencoders (image or enumerated non-canonical SMILES), gives a latent space with a better balance between SMILES similarity and a traditional molecular similarity metric. Forcing the encoder–decoder pair to trans-code between different molecular or SMILES representation indeed seems to give a latent space more tuned towards a molecular structure encoding. The latent space properties were most strongly influenced by the choice of training data and representations used for the decoder targets. The decoders trained on enumerated data also had a higher variance in produced molecules and produced SMILES during multinomial sampling. Training the decoders on enumerated data removed their tendency to only produce one canonical SMILES form of the same molecule. The larger diversity in the output may make them more relevant in de novo design approaches in drug discovery, where a balance between similarity and variance is the goal.

The improved performance when using the latent space vectors from heteroencoders for QSAR modelling, further emphasizes their increased relevance, not just being a more SMILES independent representation of the molecule, but also for a better description of the chemical space relevant for biological as well as physico-chemical properties. This should hopefully lead to more drug-discovery relevant de novo generated libraries. The increased relevance however comes at the price of greater uncertainty in the decoding, although more complex decoders seem to perform better at that metric.

## Figures and Tables

**Table 1:** Properties of the models trained on different input and output representations of the GDB-8 dataset. All values were calculated using the test dataset. Strings in the simplified molecular-input line-entry system (SMILES) notation were consideret malformed if they could not be parsed to molecules by RDKit [11].

Model	Loss	% Malformed SMILES	% Wrong Molecule	R${}^{2}$ Fingerprint Metric	R${}^{2}$ Sequence Metric
Can2Can	0.0005	0.1	0.0	0.24	**0.58**
Img2Can	0.02	0.0	8.0	0.05	0.18
Enum2Can	0.03	1.0	17.1	0.37	0.53
Can2Enum	0.18	1.7	50.3	**0.58**	0.55
Enum2Enum	0.21	2.2	66.8	0.49	0.40
Enum2Enum 2-layer	0.13	0.3	14.7	0.45	0.55

**Table 2:** Statistics on molecule generation with multinomial sampling at *t* = 1.0, *n* = 1000, GDB-8 dataset based models.

	Can2Can	Can2Enum	Enum2Enum 2-Layer
Unique SMILES	1	315	111
% Correct Mol	100	20	57
Unique SMILES for correct Mol	1	34	42
Unique Molecules	1	88	17
Average Fingerprint Similarity	1.0	0.27	0.32

**Table 3:** Reconstruction performance on the ChEMBL datasets of the different encoder/decoder configurations.

ChEMBL Model	Invalid SMILES (%)	SMILES Different from Input (%)	Wrong Molecules (%)
Can2Can	0.2	0.3	0.1
Enum2Can	9.3	42.5	36.6
Can2Enum	9.3	99.9	65.6
Enum2Enum	6.7	100	69.9

**Table 4:** Performance of the QSAR models on the held out test-set for different input data. The best performance for each metric and dataset is highlighted in bold. R^2^ is the squared correlation coefficient (closer to one is better), RMSE is the root mean square error of prediction on the test set (lower is better).

Input Type	IGC50		LD50		BCF		Solubility		MP		Average	
	R^2^	RMSE	R^2^	RMSE	R^2^	RMSE	R^2^	RMSE	R^2^	RMSE	R^2^	RMSE *
Enum2Enum	**0.81**	**0.43**	**0.68**	**0.54**	0.73	0.71	**0.90**	**0.65**	0.86	**37**	**0.80**	**0.75**
Can2Enum	0.78	0.46	**0.68**	**0.54**	**0.74**	**0.69**	0.89	0.69	0.86	**37**	0.79	0.77
Enum2Can	0.78	0.46	0.65	0.57	0.73	0.71	**0.90**	0.66	**0.87**	38	0.78	0.78
Can2Can	0.71	0.53	0.59	0.62	0.66	0.79	0.82	0.87	0.82	43	0.72	0.89
ECFP4	0.60	0.62	0.62	0.59	0.53	0.94	0.65	1.21	0.82	43	0.64	1.00

* RMSE normalized using the RMSE of ECFP4 based models before averaging.

**Table 5:** The datasets used for quantitative structure activity relationship (QSAR) modelling.

Label	Endpoint	Endpoint Values Span	Number of Molecules
BCF	Bioconcentration factor, the logarithm of the ratio of the concentration in biota to its concentration in the surrounding medium (water) [26]	−1.7 to 5.7	541
IGC50	Tetrahymena pyriformis 50% growth inhibition concentration (g/L) [27]	0.3 to 6.4	1434
LD50	Lethal Dosis 50% rats (mg/kg body weight) [28]	0.5 to 7.1	5931
MP	Melting point of solids at normal atmospheric pressure [23]	−196 to 493	7509
Solubility	log water solubility (mol/L) [16]	−11.6 to 1.6	1297

**Table 6:** Hyper parameter search space using the Tree of Parzen estimator method in Hyperopt.

Hyper Parameter	Search Space
Input dropout	0.0–0.95
Units per layer	2–1024
Kernel regularizer (L2)	0.000001–0.1
Kernel constraint (maxnorm)	0.5–6
Kernel initializer	‘lecun_uniform’ ‘glorot_uniform’, ‘he_uniform’, ‘lecun_normal’, ‘glorot_normal’, ‘he_normal’
Batch normalization	Yes (after each activation), No
Activation function	ReLU, SeLU
Dropout	0.0–0.95
Number of hidden layers	1–6
Learning rate	0.00001–0.1
Optimizer	Adam, Nadam, RMSprop, SGD

## Supplementary Materials

The following are available online at http://www.mdpi.com/2218-273X/8/4/131/s1, Figure S1, Figure S2, Figure S3.

## Abbreviations

The following abbreviations are used in this manuscript:

Conv2D	2D convolutional layer
ECFP4	Extended connectivity fingerprint with 4 bonds
GRU	Gated Recurrent Unit
LSTM	Long short-term memory
QSAR	Quantitative structure activity relationship
ReLU	Rectified liniar unit
RMSE	Root mean square error
RNN	Recurrent Neural Network
SMILES	Simplified molecular-input line-entry system
