# Supplementary material for: Improving Chemical Autoencoder Latent Space and Molecular De Novo Generation Diversity with Heteroencoders
Source: Biomolecules. 2018 Oct 30;8(4):131. doi: 10.3390/biom8040131 (PMC6316879; doi:10.3390/biom8040131)

Supplementary information for "Improving Chemical Autoencoder Latent Space and Molecular *De-novo* Generation Diversity with Heteroencoders"

Esben Jannik Bjerrum[1,*] and Boris Sattarov[2]

2018-October-17

[1]Wildcard Pharmaceutical Consulting, Zeaborg Science Center, Frødings Allé 41, 2860 Søborg, Denmark.
[2]Science Data Software LLC, 14914 Bradwill Court, Rockville, Maryland 20850, United States.
[*]Corresponding Author: esben@wildcardconsulting.dk

Figure S1: *Plot of layers used in the LSTM based auto- and heteroencoder for the GDB-8 Datasets.*

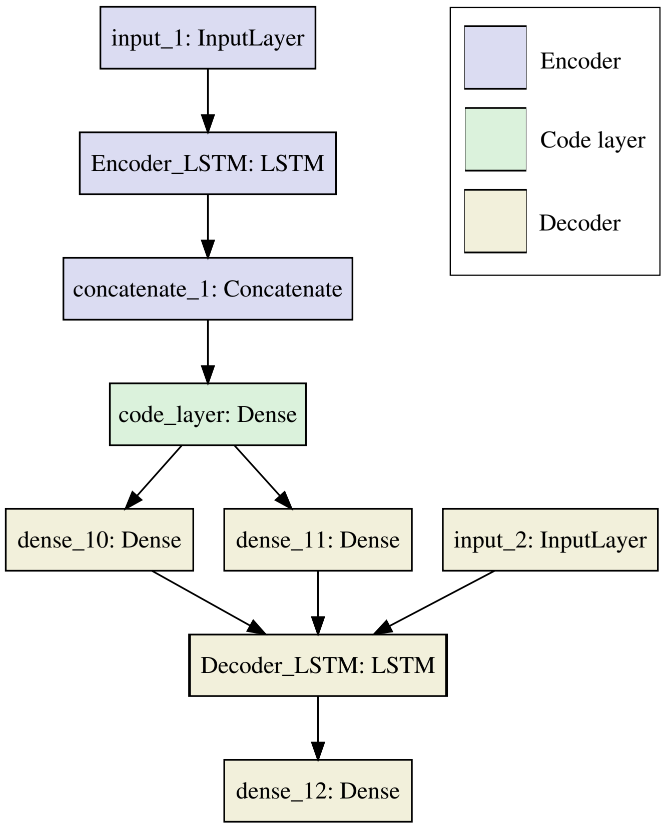

Figure S2: *Plot of the layer architecture used for the image to SMILES based model for the GDB-8 dataset. The plot is vector graphics and can be zoomed lossless.*

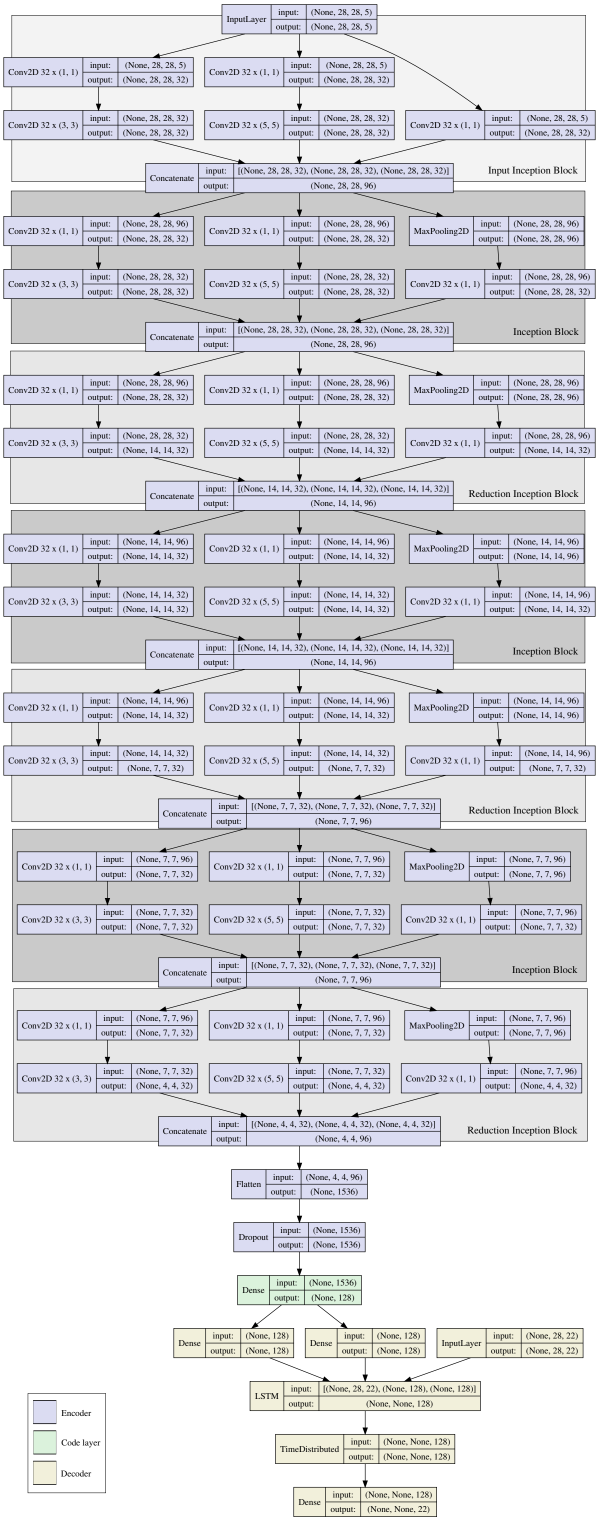

Figure S3: *Layer architecture for the auto- and heteroencoders used for the ChEMBL and QSAR datasets*

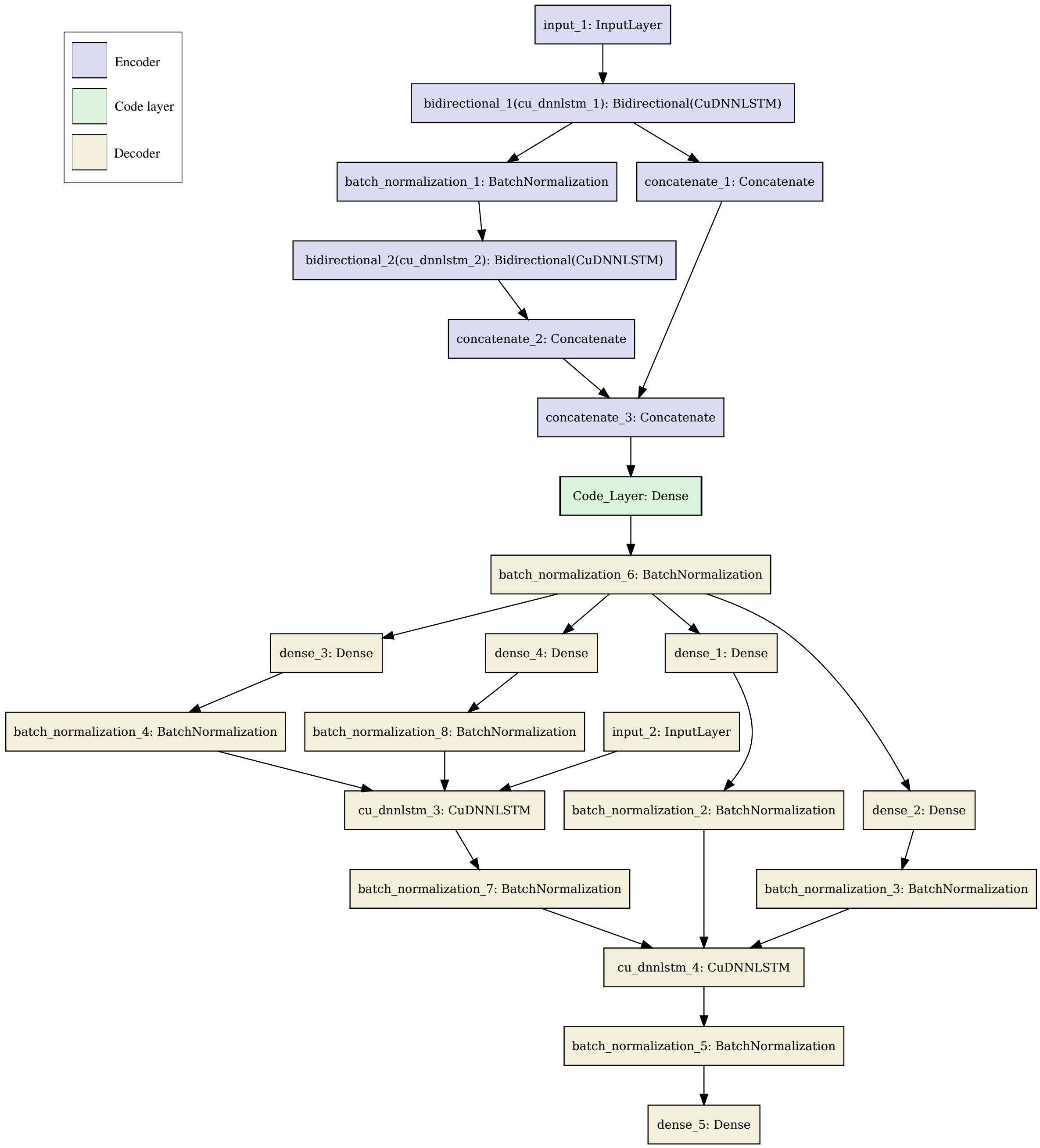

Supplement: Supplementary file 1 [file biomolecules-08-00131-s001.pdf]
